# New Insights into the Geometry and Topology of DNA Replication Intermediates

**DOI:** 10.3390/biology14050478

**Published:** 2025-04-26

**Authors:** Victor Martínez, Edith Ruiz-Díaz, Delia Cardozo, Cristian Cappo, Christian E. Schaerer, Jorge Cebrián, Dora B. Krimer, María José Fernández-Nestosa

**Affiliations:** 1 Bioinformatic Laboratory, Polytechnic School, National University of Asuncion, San Lorenzo 2111, Paraguay; 2 The Technological Research and Development Nucleus, Polytechnic School, National University of Asuncion, San Lorenzo 2111, Paraguay; 3 Department of Biomedicine, Center for Biological Research Margarita Salas, Spanish National Research Council, 28040 Madrid, Spain

**Keywords:** DNA topology, DNA replication, superhelical stress, chirality, collision events, computational biology

## Abstract

The regulation of superhelical stress, mediated by the combined action of topoisomerases and fork rotation, is crucial for DNA replication. The conformational changes during DNA replication are still experimentally challenging, mainly due to the rapid kinetics of the replication process. Here, we present the first molecular dynamics simulations of partially replicated circular DNA molecules, with stalled replication forks at both early and late stages of DNA replication. These simulations allowed us to map the distribution of superhelical stress after deproteinization. We propose a five-component model that determines the linking number difference of replication intermediates. At a thermodynamic equilibrium, the contribution of these five components was correlated to the progress of the replication forks. Additionally, we identified four types of segment collision events in replication intermediates, characterized by their geometric properties, including chirality and topological sign. The distribution of these collision events between the early and late stages of DNA replication provides new insights into the coordinated function of topoisomerases, warranting further discussion.

## Simple Summary

The topological and conformational properties of DNA replication intermediates change after deproteinization. We present the first molecular dynamics simulations of partially replicated molecules at both early and late stages of the DNA replication process. Deproteinization facilitates the distribution of superhelical stress between unreplicated and replicated regions to reach a thermodynamic equilibrium. Our simulations identified five components of superhelical stress and four types of collision events in replicating molecules. The topological sign and chirality of collision events were correlated with the progression of DNA replication.

## 1. Introduction

Dynamic changes in DNA shape are regulated by type I and type II topoisomerases that mediate inter-strand and interduplex passages, respectively. The study of these changes is part of the field of DNA topology. In all living cells, despite their shape and length, genomic DNA is organized in macrodomains (loops) that behave as closed top-ological domains [1,2]. Most of the studies on DNA topology are usually performed in small circular molecules that are called plasmids in prokaryotes.

Under physiological conditions, DNA in bacterial chromosomes and bacterial plasmids needs to be negatively supercoiled [3,4]. The process of DNA replication has a significant impact on DNA topology [5,6,7]. For topological domains, such as covalently closed circular (CCC) molecules, the progression of the replisome generates positive superhelical stress that must be removed by topoisomerases [8]. The elimination of this positive superhelical stress, by the combined action of topoisomerases and fork swiveling, induces changes that regulate the chirality and topological sign of DNA crossings, facilitating the unwinding of the double helix [2,9]. It has been shown that, during DNA replication, positive superhelical stress migrates from the unreplicated region to the replicated one, forming precatenanes [6,9]. Once replication is complete, the two daughter molecules are heavily catenated [10,11]. Sundin and Varshavsky were the first to demonstrate that the newly replicated circular DNA molecules are intertwined, forming torus-type catenanes where the sister chromatids wind around each other showing positive crossings.

DNA supercoiling refers to the underwinding (negative supercoiling) or overwinding (positive supercoiling) of the DNA double helix. Negative supercoiling facilitates the separation of parental strands during DNA replication [12,13,14]. In procaryotes, topological changes are mediated by the combined action of DNA gyrase that introduces negative supercoiling, and Topo IV that partially removes the positive supercoiling generated by the growth of the replication bubble [15,16,17]. During this dynamic process, the unreplicated region of replication intermediates (RIs) remains negatively supercoiled at the early stage of DNA replication [6]. Given the relative size of the unreplicated portion, a deproteinized RI at the early stage is expected to exhibit negative superhelical stress [18]. In contrast, at the late stage of DNA replication, the newly replicated molecules are linked, forming precatenantes with right-handed chirality and positive topological sign [18]. Consequently, a deproteinized RI at the late stage is expected to exhibit positive superhelical stress [18].

DNA superhelical stress is described by the topological invariant linking number (Lk) that measures the total number of times the parental strands are linked to each other [19]. These strands can be considered oriented curves with two geometrical properties, chirality and topological sign, that must be defined for each DNA crossing. We used the criteria proposed by Stone et al. to define the chirality of the crossings [20]. The angle between two crossing segments is characterized by the counter-clockwise rotation of the underlying strand. If the underlying strand must rotate counter-clockwise by is less than 90° to become parallel to the overlying strand, the node is called left-handed. If it must rotate more than 90˚, the node is referred to as right-handed [20]. Each perceived crossing can be assigned a topological sign based on the rotation of the overpassing strand to align with the overpassed one. If the rotation is clockwise, the sign is negative (−). If the rotation is counter-clockwise, the sign is positive (+) [2].

The Lk for a relaxed B-DNA, without superhelical stress, is called Lk0 and is calculated as Lk0 = N/10.5, where N is the total number of base pairs and 10.5 is the helical repeat [19]. Superhelical stress is measured by comparing Lk and Lk0. This is referred to as the linking number difference and is denoted by ΔLk = Lk − Lk0 [19,21]. In non-replicating molecules, Lk is expressed as the algebraic sum of twist (Tw) and writhe (Wr), while the linking number difference ΔLk is the sum of ΔTw and ΔWr [19,21,22]. Twist is a measure of the coiling of the two single DNA strands around each other in the double helix, whereas writhe quantifies the three-dimensional folding of the DNA double helix axis. DNA supercoiling can also be described by the superhelical density, defined as σ = ΔLk/Lk0 [21]. Studies on circular DNA RIs have suggested a relationship between the percentage or stage of replication and the distribution of superhelical stress, represented by the ∆Lk [23,24]. However, the geometrical properties and individual components of ∆LK in RIs remain poorly understood. The radius of gyration is another geometric descriptor that may be useful for studying conformational changes occurring during replication.

Non-replicating molecules exhibit a well-described topology. Under negative superhelical stress, they have right-handed crossings with a negative topological sign, while under positive superhelical stress, they exhibit left-handed crossings with a positive topological sign. According to Schvartzman et al., this description also applies to the unreplicated region of replicating molecules [18]. In the replicated region, precatenanes can have positive right-handed crossings (in vivo) or negative left-handed crossings (in vitro) [6,25].

The effect of supercoiling on the juxtaposition of DNA sites has been extensively analyzed before in non-replicating molecules [4,26]. Earlier studies showed the geometric characteristics of DNA juxtapositions in catenated daughter DNAs [27,28]. However, limited research has been carried out on DNA juxtapositions in partially replicated molecules. The occurrence and the geometric characteristics of DNA juxtapositions is essential for understanding the dynamic interplay among DNA topology and DNA topoisomerases. Type II topoisomerases recognize and act on specific DNA juxtapositions [29,30,31]. Two primary mechanisms identify juxtapositions in supercoiled DNA: random collision and slithering, which bring together in space two linearly distant DNA sites [29,32].

Most of the methods used to analyze DNA in vitro involve deproteinization. This means that the topology of DNA molecules examined by electrophoresis, electron microscopic or atomic force microscopy does not necessarily represent their situation in vivo [33,34,35]. The topology of replication intermediates changes significantly after deproteinization [2]. Here, we used molecular dynamics simulations on a coarse-grained model to study the distribution of the topological components of ∆Lk in partially replicated molecules. We also explored conformational changes following the deproteinization of replicating molecules by focusing on different types of collision events to be recognized as substrates for type II topoisomerases.

## 2. Materials and Methods

We considered 2 kb non-replicating circular DNA molecules and 2 kb replicating molecules at both early and late stages of DNA replication, with negative or positive superhelical stress. DNA molecules were modeled using oxDNA2, where each nucleotide contains three interaction sites responsible for stacking, electrostatic interactions, and base pairing [36,37,38,39,40]. In this model, each nucleotide is represented by a single particle, and was assigned a numerical index.

### 2.1. Modeling of Molecules

First, we generated 2 kb non-replicating CCC molecules with ∆Lk = −10. For RIs at the early stage of DNA replication, where 25% of the molecule has been replicated, the parental strand size was 1491 bp in the unreplicated region and 509 bp in the replicated region. Since Ris at the early stage of DNA replication are under negative superhelical stress in vivo, we constructed molecules with ∆Lk = −10 [18]. For Ris at the late stage of DNA replication, where 75% of the molecule has been replicated, the parental strand size was 504 bp in the unreplicated region and 1506 bp in the replicated region. Given that Ris at this stage are under positive superhelical stress in vivo, we constructed molecules with ∆Lk = +10 [18]. The tacoxDNA (Tools and Converters for oxDNA) collection of tools was used for modeling and visualization [41].

### 2.2. Molecular Dynamics Simulation

Once the model for Ris was built, a pre-equilibrium phase was carried until each region, separated by the replication forks, reached a thermodynamic equilibrium, mimicking the initial conformation before deproteinization. This initial step was performed using LAMMPS [42] to restrict particle movements at the replication forks and prevent energy exchange between the two regions. The pre-equilibrium phase was run at T = 0.1 (Lennard–Jones units) using an NVE integrator to preserve total energy. The time step was set as $10^{-4}\tau_{LJ}$ and the pre-equilibrium phase lasted to $10^{2}\tau_{LJ}$. To analyze the distribution of the ∆Lk as a function of time, we removed the fork rotation restriction from the pre-equilibrated initial configuration. The trajectory was integrated using the oxDNA2 package [36]. The system was run at a fixed temperature (20 °C) with oxDNA2 interaction types and a salt concentration of 1 M in an NVT ensemble with a John thermostat. The time step was set as $10^{-3}$ $\tau_{LJ}$, external forces were excluded, and the Verlet algorithm was used to update particles’ position and velocity.

Simulations were conducted on a computer cluster based in The Technological Research and Development Nucleus of the National University of Asuncion, and on the free webserver oxDNA.org [43]. Trajectories were visualized using oxView [44,45].

### 2.3. Topological Properties

The linking number Lk was calculated using the Gauss linking integral for two closed curves denoted as A and B [46,47,48]:

(1) $$Lk=\frac{1}{4\pi }\oint_{A}\oint_{B}\frac{\left(\vec{r_{B}}\left(t^{\prime }\right)-\vec{r_{A}}\left(t\right)\right)\cdot \vec{{dr}_{B}}\left(t^{\prime }\right)\cdot \vec{{dr}_{A}}\left(t\right)}{{\left\|\vec{r_{B}}\left(t^{\prime }\right)-\vec{r_{A}}\left(t\right)\right\|}^{3}}$$

**Figure 1:**
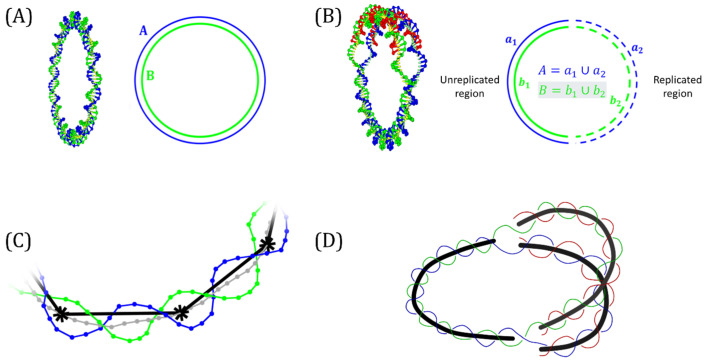
Modeling considerations. (**A**) Circular non-replicating molecule where the strands in the DNA double helix are considered as two closed curves (A and B). (**B**) Replication intermediate where each parental strand is considered as the concatenation of two curves, one corresponding to the unreplicated region ($a_{1}$ and $b_{1}$) and the other to the replicated region ($a_{2}$ and $b_{2}$). (**C**) Segment of the double helix with the midpoint curve between both DNA strands shown in gray and the reduced midpoint curve depicted in black. (**D**) Model for replication intermediates with three reduced midpoint curves (black), defined as three different sections of the partially replicated molecules with double-stranded DNA. Parental DNA strands are depicted in blue and green. Newly synthetized strands are shown in red. The midpoint curve is shown in gray and the reduced midpoint curve is depicted in black. Selected points, every 10 nucleotides, from the midpoint curve are depicted by asterisks (*).

The curve A is described by the position vector $r_{A}$ with parameter *t*, while the curve B is described by the position vector $r_{B}$ with parameter *t*′ [47,49]. In the context of DNA topology, each curve represents one strand of the DNA double helix [50,51]. Figure 1A shows a non-replicating molecule; A and B are defined as piecewise smooth curves and each straight segment has a start and end point at the position of particles (nucleotides) with indices I and i + 1, respectively. The number of segments in each curve was equal to the number of base pairs in the modeled molecule. Evaluating the integral between segment i of one curve and segment j of the other gives a solid angle element ${Ω}_{ij}$, and the sum of all the index pairs of the complete molecule gives the Lk [47,49]. Figure 1B corresponds to a replicated intermediate, each parental strand is separated into two sections: one in the unreplicated region and the other in the replicated portion, so that both curves can be represented as $A=a_{1}\cup a_{2}$ and $B=b_{1}\cup b_{2}$. The Lk can be calculated as the sum of three terms:

(2) $$Lk=\oint_{A}\oint_{B}dΩ=\int_{a_{1}}\int_{b_{1}}dΩ+\int_{a_{1}/b_{1}}\int_{b_{2}/a_{2}}dΩ+\int_{a_{2}}\int_{b_{2}}dΩ$$

The calculation of each solid angle ${Ω}_{ij}$, defined by the straight segments, i and j can be represented as an entry in a square contribution matrix. Each of these entries contains information about the topological charge that corresponds to the magnitude and the topological sign. The size of this matrix was n × n, where n is the length, or number the bp, of the DNA molecule.

We considered a collision event when the distance between the center of mass of two base pairs in the double helix was ≤10 nm. This condition applied only for nucleotide indices with a distance greater than the persistence length. The minimum distance (10 nm) corresponds to the average size of proteins that interact with two DNA sites simultaneously [29].

Collision events were detected by computing the midpoint of opposite sides for each base pair along parental strands. The set of midpoints determined the midpoint curve. To avoid the noise from oversampling, a reduced midpoint curve was obtained by selecting points at every 10 nucleotides from the midpoint curve (near a complete turn of the double-helix). The construction of the midpoint and the reduced midpoint curves is shown in Figure 1C. For RIs, we considered three reduced midpoint curves (Figure 1D). Considering the segments defined by points $\vec{r_{A}}\left(i\right)$ and $\vec{r_{A}}\left(i+1\right)$ and points $\vec{r_{B}}\left(j\right)$ and $\vec{r_{B}}\left(j+1\right)$, we calculated the distance between these two segments as follows:

(3) $$d=\frac{\left|\left(\vec{r_{A}}\left(i\right)-\vec{r_{B}}\left(j\right)\right)\cdot \left(\vec{u}\times \vec{v}\right)\right|}{\left|\vec{u}\times \vec{v}\right|}$$

where $\vec{u}=\vec{r_{A}}\left(i+1\right)-\vec{r_{A}}\left(i\right)$ and $\vec{v}=\vec{r_{B}}\left(j+1\right)-\vec{r_{B}}\left(j\right)$.

The calculation of radius of gyration was carried out by applying the following:

(4) $$R_{g}=\sqrt{\frac{\sum_{k}^{N}{\left\|\vec{r}\left(k\right)-{\vec{r}}_{CM}\right\|}^{2}}{N}}$$

where N is the total number of nucleotides in the molecule, k refers the index of the nucleotide and ${\vec{r}}_{CM}$ is the center of mass of the system [52,53].

## 3. Results

### 3.1. Distribution of Superhelical Stress After Deproteinization

**Figure 2:**
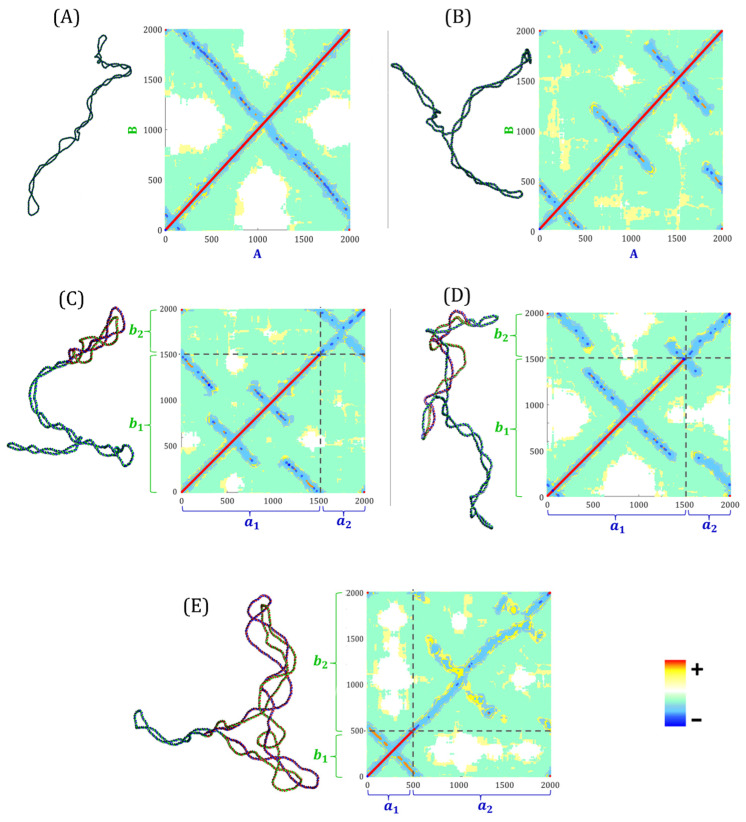
Correspondence between DNA conformation and the Lk contribution matrix. Typical conformations of the simulated CCC molecules and their contribution matrix of the Gauss linking integral for (**A**) non-replicating molecules with simple plectonemic supercoiling. (**B**) Non-replicating molecules with three plectonemic branches. (**C**) Early-stage replication intermediate showing plectonemes of precatenanes. (**D**) Early-stage replication intermediate showing the wrapping of the parental and daughter strands. (**E**) Late-stage replication intermediate with plectonemes in the unreplicated region and plectonemes of precatenanes in the replicated region. Parental strands are shown in blue and green, while newly synthetized DNA is red. The horizontal and vertical axes refer to each parental strand: A and B for non-replicating molecules; and $a_{1}$, $a_{2}$, $b_{1}$ and $b_{2}$ for replication intermediates. The color key bar denotes the topological charge.

Typical conformations of non-replicating molecules with ∆Lk = −10, in the equilibrium state, and their corresponding Lk contribution matrices are shown in Figure 2A,B.

The positive topological charge of the double helix accumulates along the main diagonal, while secondary diagonals correspond to the negative topological charge of supercoiling. In the case of the unbranched plectonemic non-replicating molecule shown in Figure 2A, the contribution matrix presents a single secondary diagonal corresponding to the negative contribution of the supercoiling. Figure 2B shows a branched plectonemic non-replicating molecule. Three secondary diagonals, corresponding to the negative contributions of the three plectonemic branches, are clearly distinguished. In Supplementary Figure S1, we present the case of a non-replicating molecule with negative supercoiling and four plectonemic branches.

Figure 2C,D shows typical conformations of RIs at the early stage of replication, with ∆Lk = −10, in the equilibrium state, and their respective Lk contribution matrices. Each matrix is divided into four submatrices defined by the segments $a_{1}$, $a_{2}$ and $b_{1}$, $b_{2}$ of a RI, where $a_{1}$ and $b_{1}$ represent the unreplicated portion, and $a_{2}$ and $b_{2}$ the replicated portion (see Figure 1B). The largest square submatrix (bottom left) corresponds to the contributions of segments $a_{1}$ and $b_{1}$ from the unreplicated region. The two rectangular submatrices (top left and bottom right) correspond to the contributions of $a_{1}$, $b_{2}$ and $a_{2}$, $b_{1}$. The smallest square submatrix (top right) corresponds to the contribution of segments $a_{2}$ and $b_{2}$. In both matrices (Figure 2C,D), the main diagonal of the largest square submatrix, represents the positive topological charge of the double helix. Figure 2C shows a molecule at the early stage of replication with plectonemic branches. The largest square submatrix shows three secondary diagonals corresponding to the negative contributions of the plectonemic branches. Within the smallest square submatrix, the main diagonal corresponds to precatenanes with a negative topological charge, while the secondary diagonal corresponds to a plectoneme of precatenanes. These results indicate that supercoiling of the unreplicated region and plectonemes of precatenanes are visualized as secondary diagonals in the contribution matrix. Figure 2D shows an RI at the early stage with precatenates in the replicated region. The absence of a secondary diagonal in the smallest square submatrix corresponds to the lack of plectonemes of precatenanes. Unlike the case shown in Figure 2C, the two rectangular submatrices show contributions between the unreplicated and the replicated regions. The wrapping of both regions is shown in the corresponding conformation.

Figure 2E shows the typical conformation of an RI at the late stage of replication, with ∆Lk = +10, in the equilibrium state, along with its Lk contribution matrix. The largest square submatrix (top right) corresponds to the contributions of segments $a_{2}$ and $b_{2}$. The two rectangular submatrices (top left and bottom right) correspond to the contributions of segments $a_{1}$, $b_{2}$ and $a_{2}$, $b_{1}$. The smallest square submatrix (bottom left) corresponds to contributions between segments $a_{1}$ and $b_{1}$. This submatrix shows a main diagonal, representing the positive topological charge of the double helix, and a secondary diagonal, corresponding to the positive supercoiling in the unreplicated region. In the largest square submatrix, the main diagonal corresponds to the positive contributions of the precatenanes, while secondary diagonals correspond to positive plectonemes of precatenanes. Our results confirm that secondary diagonals are associated with plectonemic forms in in both non-replicating and partially replicated molecules. Overall, our contribution matrices show a dynamic distribution of superhelical stress during the replication process. In addition to ∆Tw and Wr, other topological components contribute to ∆Lk in partially replicated molecules: precatenanes, wrapping between parental and daughter strands, and plectonemes of precatenanes.

### 3.2. Temporal Evolution of the Topological Components of ∆LK in Deproteinized Replication Intermediates

Based on the results obtained from the Lk contribution matrices, we identified five different topological components of ∆Lk in RIs: the ∆Tw of the unreplicated region (Component 1), the Wr of the unreplicated region (Component 2), the wrapping between the strands of the unreplicated and replicated regions (Component 3), the precatenation (Component 4), and the plectonemes of precatenanes (Component 5). We studied the temporal evolution of these five components of ∆Lk for early and late stages of replication, from deproteinization to thermodynamic equilibrium. Each simulation was divided into six parts to observe the gradual distribution of torsional stress. In all cases, the final conformation of each part was used as the initial conformation of the next one. The cumulative time span covered by all simulations was ~2 × $10^{7}\tau_{LJ}$ (~1.5 ms), corresponding to ~2 × $10^{9}$ time steps.

**Figure 3:**
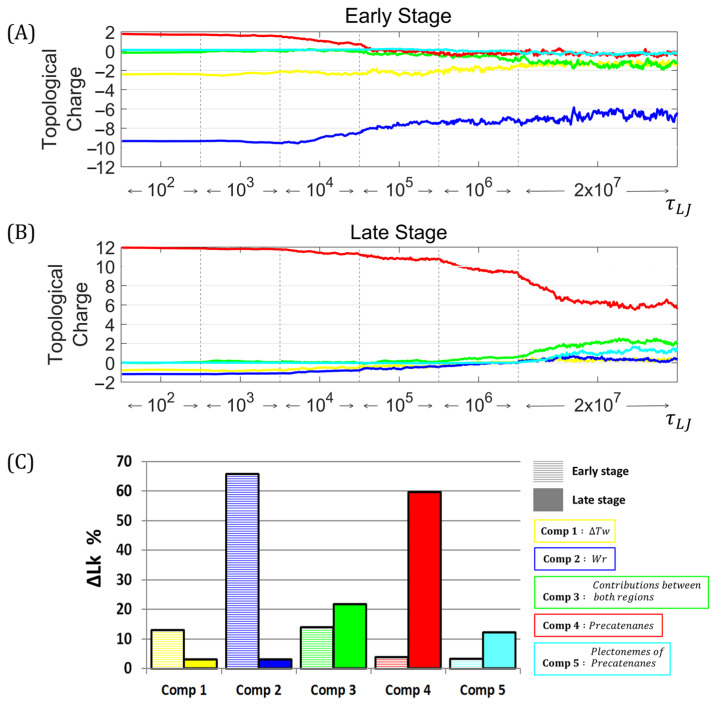
Temporal distribution of ∆Lk after the deproteinization of replication intermediates. Average semi-logarithmic time progression of ∆Lk contribution after deproteinization of replication intermediates. Each graphic corresponds 10 independent simulations. (**A**) Early-stage replication intermediate with ∆Lk = −10. (**B**) Late-stage replication intermediate with ∆Lk = +10. (**C**) Percentage distribution of ∆Lk in the equilibrium state for early (striped bars) and late (solid bars) stages. The ∆Tw of the unreplicated region (Component 1) is represented in yellow. The Wr of the unreplicated region (Component 2) is blue. The contribution of the wrapping between parental and daughter strands (Component 3) is green. The contribution of precatenanes (Component 4) is red. The contribution of plectonemes of precatenanes (Component 5) is cyan.

For RIs at the early stage, the initial conformation, corresponding to the in vivo condition, had a topological charge of −12 in the unreplicated region and +2 in the replicated portion. The average semi-logarithmic time evolution (Figure 3A), corresponding to 10 independent simulations, showed an initial period of gradual change, followed by a high rate of changes occurring at ~${0.5\times 10}^{4}\tau_{LJ}$, especially for Components 2 and 4. Components 1 and 5 remained stable, with a negative topological charge throughout the simulation. Component 3 showed a shift toward negative values occurring at $10^{6}\tau_{LJ}$. Molecules reached a thermodynamic equilibrium during the last $10^{7}\tau_{LJ}$. The topological charge of Components 1, 2 and 3 was negative, while for Components 4 and 5, it was close to zero. The average final conformation, corresponding to the in vitro condition, had a topological charge of −7.88 in the unreplicated region, −0.71 in the replicated potion, and −1.40 shared between both regions.

For RIs at the late stage, the initial conformation, corresponding to the in vivo condition, had a topological charge of −2 in the unreplicated region and +12 in the replicated region. The average semi-logarithmic time evolution (Figure 3B), corresponding to 10 independent simulations, showed an initial period with a low change rate, followed by a shift at $10^{6}\tau_{LJ}$, especially for Components 2 and 4. Components 1 and 3 remained close to zero throughout the simulation, acquiring a positive topological charge at ${\sim 1.1\times 10}^{6}\tau_{LJ}$. Molecules reached thermodynamic equilibrium in the last $10^{7}\tau_{LJ}$. The topological charge of Components 1 and 2 was close to zero, while for Components 3, 4 and 5 was positive. The final conformation, corresponding to in vitro condition, had a topological charge of +0.61 in the unreplicated region, +7.20 in the replicated potion, and +2.19 shared between both regions.

In the equilibrium state, the ∆LK of the RIs at the early stage was mainly distributed in Components 1, 2 and 3, with an inverse relationship between Components 2 and 3. The ∆LK of the RIs at the late stage of replication was mainly distributed in Components 3, 4 and 5, with an inverse relationship between Components 3 and 4.

Figure 3C shows a comparison of the average contribution of each component of the ∆Lk in the equilibrium state. At the early stage of replication, Components 1 and 2, corresponding to the unreplicated region, represented 78.82% of the ∆Lk. At the late stage of replication, Components 4 and 5, corresponding to the replicated region, represented 71.97% of the ∆Lk. Component 3, corresponding to the wrapping between the strands of both regions, represented 14.04% and 21.88% for early and late stages, respectively. We, therefore, concluded that the supercoiling of the unreplicated region and wrapping between parental and daughter strands are the main components of superhelical stress at the early stage of replication. For late-stage RIs, the primary components are precatenanes and their plectonemic conformations.

### 3.3. Dynamics of Collision Events in Deproteinized Replication Intermediates

**Figure 4:**
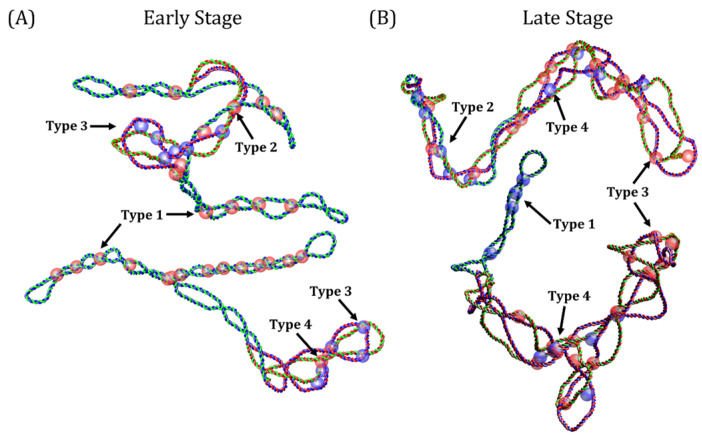
Collision events in deproteinized replication intermediates. Types of collision events identified in the equilibrium state. (**A**) Early-stage replication intermediates. (**B**) Late-stage replication intermediates. Parental strands are depicted in blue and green, newly synthetized strands in red. Examples of different types of collision events are indicated with numbers (1–4). Right-and left-handed collisions are represented by red and blue spheres with a radius of 10 nm, respectively.

We investigated collision events in molecules at the early and late stages of replication after deproteinization. A collision event occurs when the distance between two distal sites of a DNA double helix is ≤10 nm, the average size of proteins interacting with two DNA sites simultaneously [16]. We applied this condition to RIs and selected the local minima that satisfy the minimum distance (Supplementary Figure S2). In this way, we were able to identify four types of collisions events, which were classified as follows: Type 1, collisions between two distal segments of the unreplicated region; Type 2, collisions between segments of the unreplicated and replicated regions; Type 3, collisions that occur within the precatenanes, and Type 4, collisions that occur within the plectonemes of precatenanes. A collision event has the same topological properties of a DNA crossing, allowing the determination of its chirality and topological sign. Figure 4 show examples of the different types of collisions and their chirality for deproteinized RIs at the early (Figure 4A) or late stages (Figure 4B) of replication.

**Figure 5:**
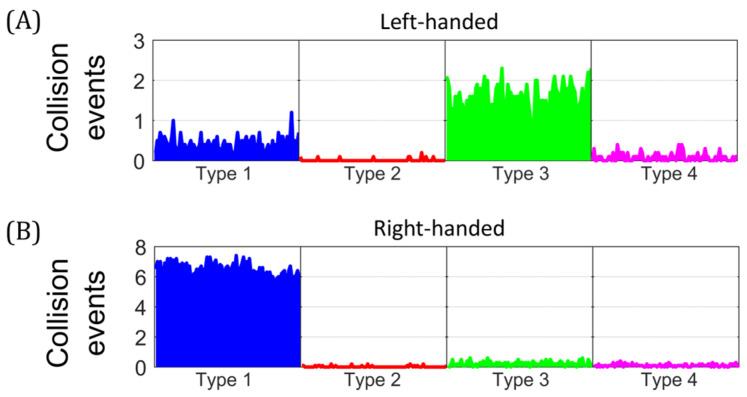
Mean temporal traces of collision events for early-stage replication intermediates. Number of collisions events in partially replicated molecules with ∆Lk = −10 in the equilibrium state. (**A**) Left-handed collision events. (**B**) Right-handed collision events. Average time progression from 10 independent simulations.

We compared the occurrence of different types of collision events and their chirality for both early and late stages, in the equilibrium state. Figure 5 shows the average of 10 independent simulations for RIs at the early stage with negative superhelical stress (∆Lk = −10). Collisions were classified, according to their chirality, into left-handed (Figure 5A) and right-handed (Figure 5B). Type 1 was the most abundant, with mean values of 0.42 and 6.42 for left- and right-handed collisions, respectively. This was followed by Type 3, with mean values of 1.57 and 0.23 for left- and right-handed collisions, respectively. Types 2 and 4 collisions had low occurrence, with values ≤ 0.09. These results showed that at the early stage of replication, negatively supercoiled molecules predominantly exhibited right-handed collision events within the larger unreplicated region. In contrast, left-handed collisions, located within the precatenanes, predominated in the smaller replicated region.

**Figure 6:**
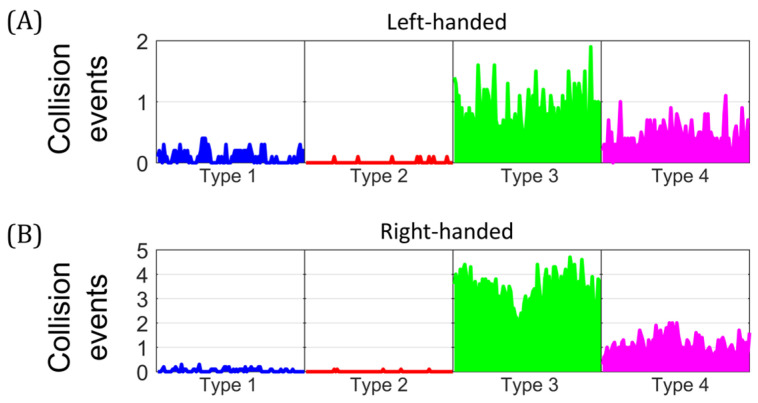
Mean temporal traces of collision events for late-stage replication intermediates. Number of collisions events in partially replicated molecules with ∆Lk = +10 in the equilibrium state. (**A**) Left-handed collision events. (**B**) Right-handed collision events. Average time progression from 10 independent simulations.

Figure 6 shows the average of 10 independent simulations for RIs at the late stage with positive superhelical stress (∆Lk = +10). Collision events were classified according to their chirality into left-handed (Figure 6A) and right-handed (Figure 6B). Type 3 was the most abundant, with mean values of 0.83 and 3.25 for left- and right-handed collisions, respectively. This was followed by Type 4, with mean values of 0.43 and 0.97 for left- and right-handed collisions, respectively. Types 1 and 2 had low occurrence, with values ≤ 0.05. These results showed that RIs at the late stage had mainly right-handed collisions, located in the replicated region, while left-handed collisions predominated in the unreplicated portion. Supplemental Figure S3 shows additional initial conformations, corresponding to the in vivo situation, for RIs at the early and late stages. Supplemental Figures S4 and S5 show the temporal evolution of collision events after deproteinization. A percentage distribution of the collisions events in the equilibrium state is shown in Supplemental Table S1. To summarize, right-handed collisions predominated in both stages of the replication and were mainly located in the largest region of the molecule. In comparison, left-handed collisions predominated in the smallest region.

**Figure 7:**
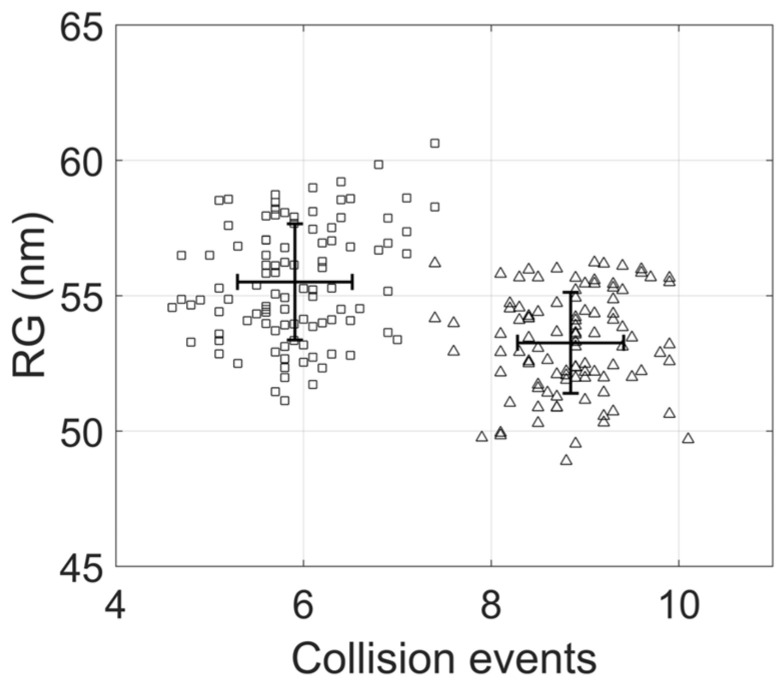
Radius of gyration in deproteinized replication intermediates. The radius of gyration and the number of collision events for molecules at early (Δ) and late stages (□) of replication.

The radius of gyration is a key geometric descriptor in DNA topology that depends on superhelical stress and different forms of topological complexity, such as DNA knotting [22,34,52,54,55,56]. To characterize the geometrical changes related to the progression of DNA replication, we compared the values of the radius of gyration with the number of collision events in replicating molecules at the early and late stages of replication. Figure 7 shows the radius of gyration and the number of collision events in the equilibrium state. RIs at the late stage exhibited a higher radius of gyration and a lower number of collision events than molecules at the early stage of replication. For both cases, the dispersion in collision events was relatively low, resulting in the absence of the overlap between their number of collision events. However, the radius of gyration exhibited significant dispersion, leading to considerable overlap between early and late stages.

## 4. Discussion

The structure of circular DNA RIs has been studied in detail using both gel electrophoresis and electron microscopy [23,25,57,58]. Studies on circular DNA replication have shown that once replication is completed, the two daughter molecules exhibit positive right-handed catenation [59,60]. These catenanes originate from precatenanes formed in vivo during DNA replication by rotation of the replication forks, which allows the migration of torsional stress from the unreplicated region to the replicated portion [6,18]. Ullsperger et al. referred to RIs with supercoiling and precatenanes as “butterfly forms” [23]. Cebrian et al. demonstrated that in bacterial plasmids precatenanes form as replication progresses before termination [6]. On the other hand, experiments on budding yeast suggested that torsional stress migrates to the replicated region only during the final stages of DNA replication [61].

To our knowledge, this is the first molecular dynamics simulation study to reveal the dynamic DNA topology of partially replicated molecules. We constructed RIs at the early and late stages of replication, where 25% and 75% of DNA has been replicated, respectively. In each case, the molecules were endowed with physiological levels of superhelical stress, mimicking the in vivo conditions just before deproteinization. Based on Lk contribution matrices for molecules at the early and late stages, we identified five components of ∆Lk for RIs in the equilibrium state. In the early stage of DNA replication, Wr was the major contributor to ∆Lk. Conversely, precatenanes became the primary determinant component of ∆Lk in the late stage. These observations suggests that the distribution of ∆Lk components in partially replicated molecules changes during the replication process and depends on the relative sizes of the unreplicated and replicated regions. This result is consistent with previous studies based on energy considerations and the distribution of superhelical stress between the unreplicated and replicated regions [23,24].

In the initial conformations, right-handed chirality predominated throughout the RI at both, the early and late stages. This corresponds to the conformation of partially replicated molecules in vivo, harboring negative right-handed crossings in the unreplicated region and positive right-handed precatenanes in the replicated region [2,18]. On the other hand, we found that in the equilibrium state, corresponding to the in vitro condition, the predominant chirality was opposite in both regions of RIs at the early and late stages. Right-handed collisions, the most frequent in both stages, were mainly located in the largest region of the molecule, while left-handed collisions predominated in the smallest portion. In both cases, there was a relation between the relative size of the unreplicated and replicated regions and the chirality of the collision events. Our results support the proposal by Schvartzman et al. that the topology of RIs changes significantly after deproteinization [18].

In molecules at the early stage of replication, the chirality of precatenanes crossings in the replicated region changes from right- to left-handed. In molecules at the late stages of replication, the chirality of supercoil crossings in the unreplicated region changes from right- to left-handed. Therefore, the topology of RIs after deproteinization does not accurately reflect the in vivo situation. These changes may have important experimental implications when RIs are analyzed by two-dimensional gel electrophoresis or electron microscopy and may also affect topoisomerase processivity during in vitro assays.

Bacterial type II topoisomerases are essential enzymes that regulate DNA topology, remove tangles, and maintain the correct structure of the genome. DNA gyrase is responsible for maintaining negative supercoiling of bacterial chromosome, while topoisomerase IV acts in disentangling daughter chromosomes following replication. The enzymes have a wide variety of possible substrates [62]. The main mode of activity of DNA gyrase is the introduction of negative supercoiling, resulting in a net change in Lk of −2, at the cost of consuming ATP [63,64,65,66,67,68,69]. This catalytic mechanism involves a wrapping movement to confront the T and G segments, provided that the DNA is subjected to low forces and torques [64]. In the in vivo situation, corresponding to the initial conformation, of RIs at the early stage, DNA gyrase introduces negative right-handed crossings in the unreplicated region. However, immediately after deproteinization, the excess superhelical stress from these right-handed crossings migrates to the replicated region, promoting the formation of negative left-handed precatenanes. High levels of negative superhelical stress in early-stage RIs result in an accumulation of left-handed crossings in the replicated region, generating high torsional stress. At forces and torques that inhibit DNA wrapping, DNA gyrase captures a distal T segment, leading to the relaxation of two left-handed crossings [64].

At equilibrium state, corresponding to the in vitro situation, late-stage RIs with positive ∆Lk exhibit left-handed crossings in the unreplicated region and right-handed crossings in the replicated region. Left-handed crossings are the ideal substrate for DNA gyrase, and long-term action will result in total relaxation of the unreplicated region. In vitro experiments have demonstrated that, in addition to its role in the introduction of negative supercoiling, DNA gyrase can relax precatenanes in partially replicated plasmids [70].

Topo IV unlinks catenated chromosomes before cell division and relaxes positive supercoils generated during DNA replication [71,72,73]. In vivo experiments by Helgesen et al. suggested that Topo IV primarly acts behind the replication fork, in the replicated region, eliminating precatenanes [74]. Studies on braided molecules have shown that precatenanes can buckle to form left-handed plectonemes of precatenanes when the density of precatenanes is sufficiently high [35,75]. Marko predicted buckling to occur at a value around 0.045 [76]. Our molecular dynamic simulations of RIs confirmed that buckling occurs at the early stage of replication. Relaxation of right-handed precatenanes could therefore occur inside a left-handed plectoneme of precatenanes like those shown in Figure 2D. For precatenanes below their buckling threshold, Topo IV could remove right-handed precatenanes, but at a slower rate [77].

At equilibrium state, corresponding to in vitro condition, Topo IV would predominantly resolve left-handed crossings in the replicated region and right-handed crossings in the unreplicated portion. In vitro experiments have demonstrated that Topo IV can eliminate left-handed crossings on a partially replicated plasmid [70]. Despite geometrical considerations, some studies have demonstrated that bacterial type II topoisomerases can recognize specific DNA sequences as cleavage sites and convert them into gate-DNA sites. Arnoldi et al. suggested that specific sequences of bacterial DNA can be recognized by type II topoisomerases [30]. The results obtained by Morgan et al. using the SHAN-seq sequencing technique, found numerous cleavage sites for DNA gyrase and Topo IV in prokaryotic DNA, indicating that topoisomerases recognize specific sequences as gate-DNA sites [31]. They also noted that although the chirality of crossings alters the frequency at which cleavages are established, it has no effect on the selection of the gate sites.

The radius of gyration is a key geometric descriptor that influences DNA behavior in microfluidic devices and separation processes. Experimental results demonstrate that replication progression is associated with a decrease in electrophoretic mobility [6]. Our simulations showed that the radius of gyration of molecules at the late stage is larger than that of molecules at the early stages. This difference may be linked to the total number of collision events observed at both stages. Future research should incorporate external forces to represent the electric field and a fibrous medium to model electrophoresis in agarose gels.

The results presented here may provide a new mathematical approach to understanding the distribution of the linking number difference in replicating molecules. Significant discrepancies, regarding chirality and the topological sign of collision events were observed in RIs after deproteinization. Additional studies are needed to fully understand the influence of topological and geometrical factors on DNA replication and the role of topoisomerases throughout this process.

### Limitations

Our study has certain limitations that should be acknowledged. First, the observations made on simulated bacterial plasmids, which correspond to relatively small topological domains, may not accurately reflect the distribution of torsional stress occurring within the larger loops or compartments of eukaryotic chromosomes. Second, the topological changes described here were analyzed on simulated partially replicated DNA molecules after removal of associated proteins, corresponding to in vitro condition used in electrophoresis, electron microscopy, or atomic force microscopy. Although our findings provide valuable structural insights, studying DNA topology in vitro does not necessarily represent the dynamic interactions of supercoiled DNA within the cellular environment. Therefore, extrapolations from in vitro to in vivo conditions should be made with caution, and further studies are needed to validate these findings in physiological contexts.

## 5. Conclusions

Here, we report the first molecular dynamics simulation of partially replicated molecules with forks stalled at different sites before termination. We characterized the topological components of superhelical stress and their distribution during DNA replication. For early-stage RIs, were only 25% of the molecule is replicated, most of the superhelical stress takes the form of supercoils in the unreplicated region and wrapping of parental and daughter strands. In contrast, for late-stage RIs, where 75% of the molecules have already been replicated, superhelical stress is predominantly accommodated by precatenanes and their plectonemic forms. The geometrical and topological properties of RIs change significantly in vitro compared to in vivo. In deproteinized molecules, the free swiveling of replication forks allows the distribution of superhelical stress between the unreplicated and replicated regions to reach a state of thermodynamic equilibrium. Our analysis revealed a strong correlation between DNA replication progression and the distribution and chirality of collision events. Throughout replication stages, right-handed collisions predominated the larger region, while the smaller region exhibited a chirality shift from right- to left-handed.

## Data Availability

The original contributions presented in this study are included in the article and the Supplementary Materials. Further inquiries can be directed to the corresponding author.

## Supplementary Materials

The following supporting information can be downloaded at: https://www.mdpi.com/article/10.3390/biology14050478/s1, Figure S1: Lk contribution matrix of a non-replicating molecule with four plectonemic branches; Figure S2: Selection of collision events in a non-replicating molecule; Figure S3: Segment collision events in the initial conformations of replication intermediates; Figure S4: Mean temporal traces of collision events for replication intermediates at the early stage; Figure S5: Mean temporal traces of collision events for replication intermediates at the late stage; Table S1: Percentage distribution of the different types of collision events.

## Abbreviations

The following abbreviations are used in this manuscript:

CCC	Covalently closed circular
RI	Replication intermediate
Lk	Linking number
∆Lk	Linking number variation
Tw	Twist
∆Tw	Twist variation
Wr	Writhe
ΔWr	Writhe variation
