# Supplementary material for: New Insights into the Geometry and Topology of DNA Replication Intermediates
Source: Biology (Basel). 2025 Apr 26;14(5):478. doi: 10.3390/biology14050478 (PMC12109278; doi:10.3390/biology14050478)
Supplement: Supplementary file 1 [file biology-14-00478-s001.zip › biology-3533499-supplementary.pdf]

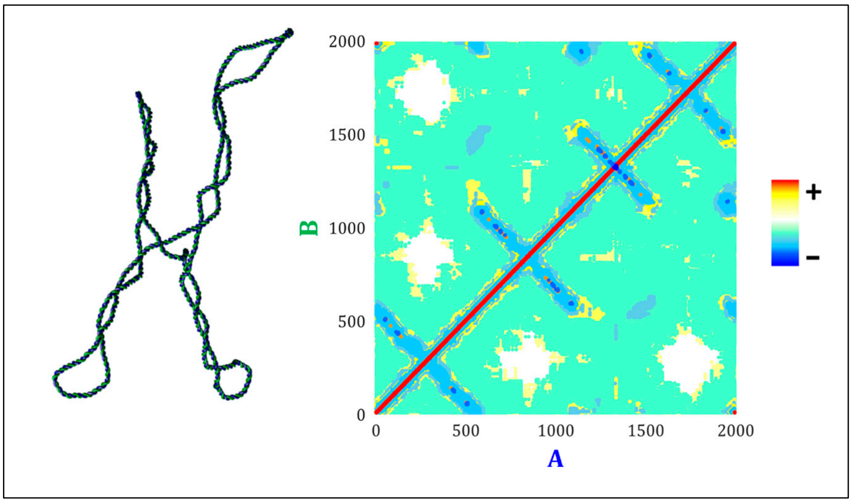

Figure S1. Lk contribution matrix of a non-replicating molecule with four plectonemic branches. Conformation of a CCC molecule with four branches and the plot of the contribution matrix of the Gauss linking integral. Horizontal and vertical axes refer to each strand: A and B. The color key bar denotes the topological charge.

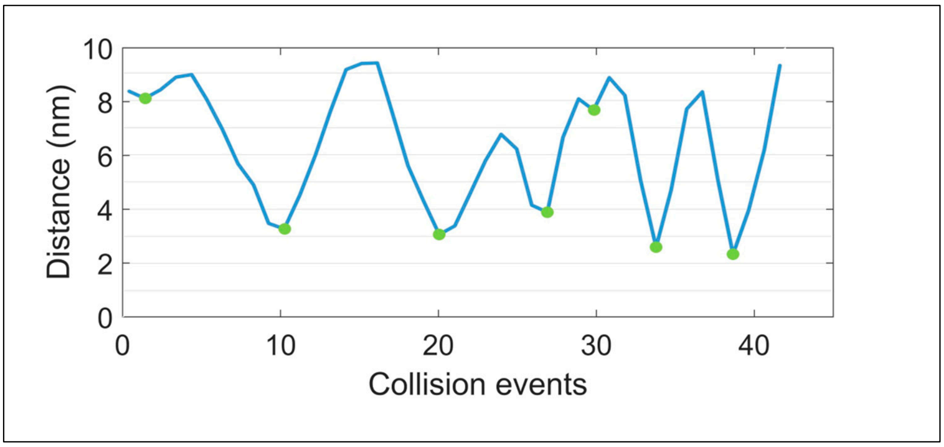

Figure S2. Selection of the collision events in a non-replicating molecule. Graphic showing collision events that are below the threshold distance of 10 nm. Selected collision events that correspond to the local minima are depicted in green.

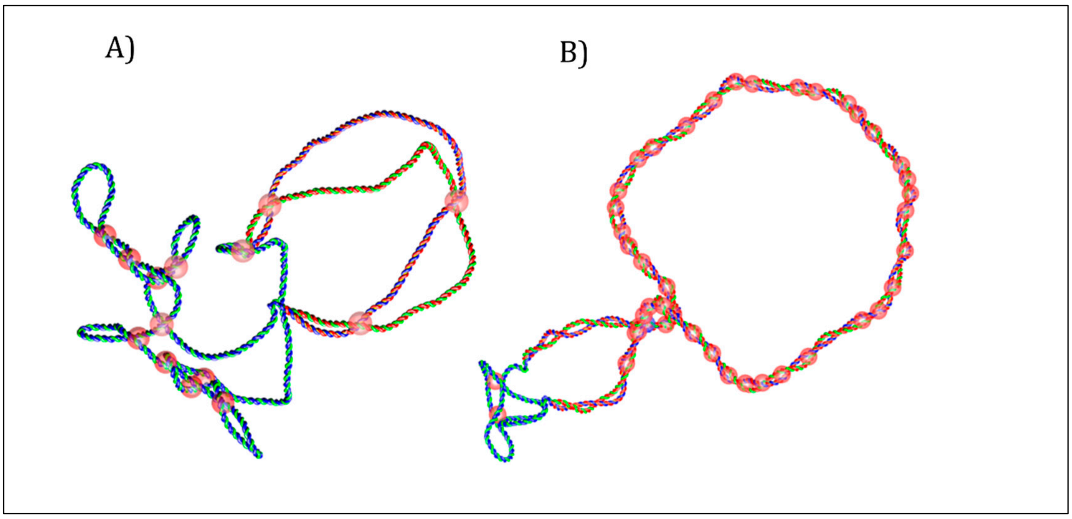

Figure S3. Segment collision events in the initial conformations of replication intermediates. A) Early-stage RI. B) Late-stage RI. Parental strands are depicted in blue and green, and newly synthesized strands in red. Right-handed collision events are represented by red and spheres with a radius of 10 nm.

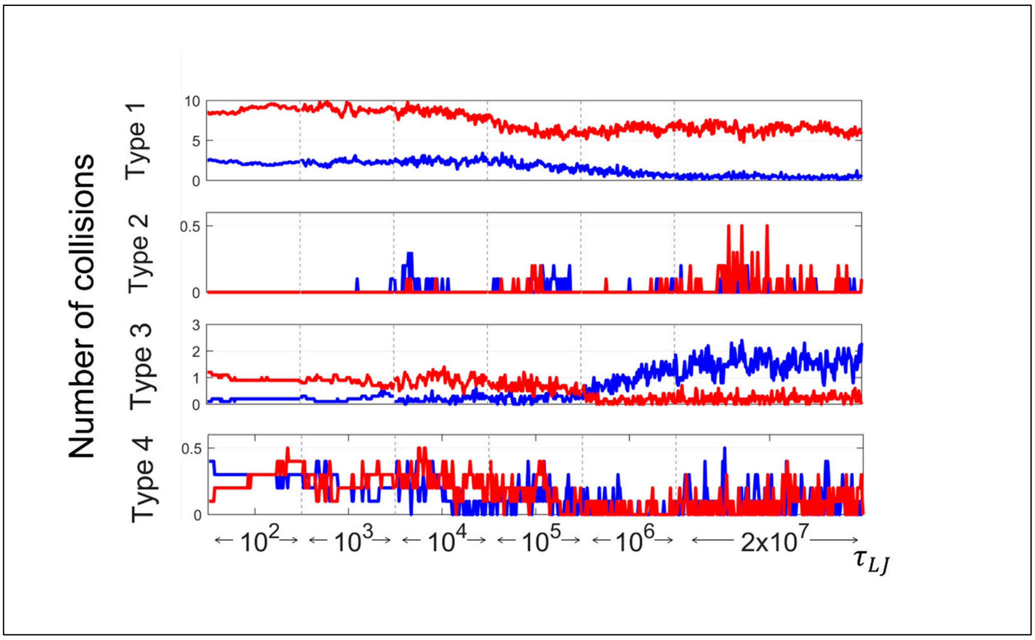

Figure S4. Mean temporal traces of collision events for early-stage replication intermediates. Average semi-logarithmic time progression from 10 independent simulations. Number of collision events in partially replicated molecules with ΔLk = -10 in the equilibrium state. Left-handed collisions are shown in blue, and right-handed collisions in red.

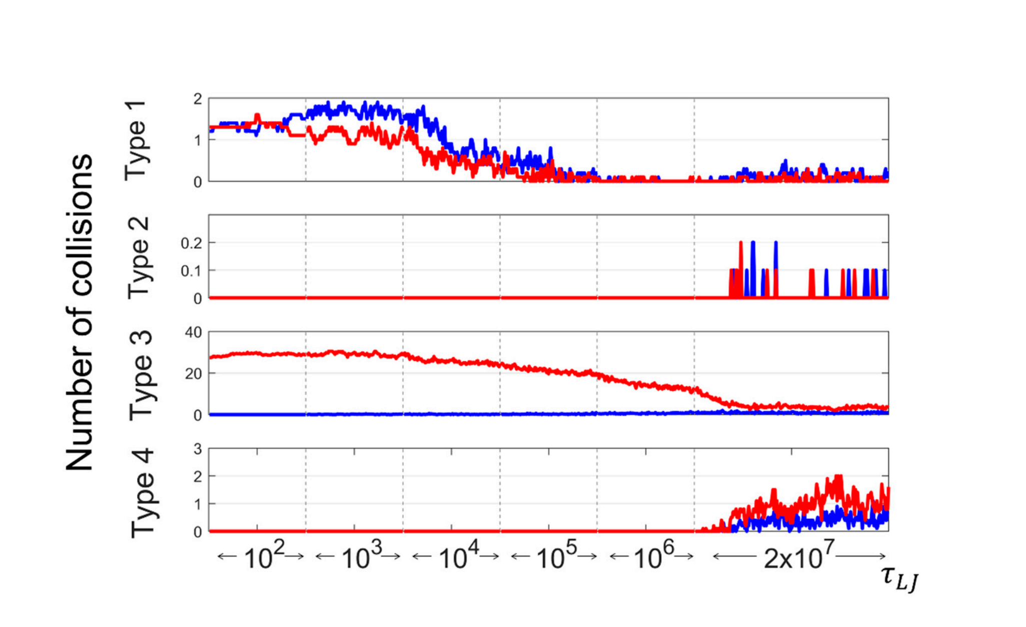

Figure S5. Mean temporal traces of collision events for late-stage replication intermediates. Average semi-logarithmic time progression from 10 independent simulations. Number of collision events in partially replicated molecules with ΔLk = +10 in the equilibrium state. Left-handed collisions are shown in blue and right-handed collisions in red.

Table S1. Percentage distribution of the different types of collision events.

	**Early stage**				**Late stage**			
**|ΔLk| = 10**	**RH−**	**RH+**	**LH−**	**LH+**	**RH−**	**RH+**	**LH−**	**LH+**
Type 1 collision	72.2	0.2	4.6	0.5	0	0.5	0	0.9
Type 2 collision	0.5	0.1	0.1	0	0	0.1	0.0	0.2
Type 3 collision	2.1	0.3	17.6	0	0.1	68.2	1.2	13.2
Type 4 collision	0.8	0	1.0	0	0.3	10.4	0.6	4.3
